# Supplementary material for: Common diseases alter the physiological age-related blood microRNA profile
Source: Nat Commun. 2020 Nov 24;11:5958. doi: 10.1038/s41467-020-19665-1 (PMC7686493; doi:10.1038/s41467-020-19665-1)

Common diseases alter the physiological age-related blood microRNA profile

Tobias Fehlmann[1], Benoit Lehallier[2], Nicholas Schaum[2], Oliver Hahn[2], Mustafa Kahramann[1], Yongping Li[1], Nadja Grammes[1], Lars Geffers[3], Christina Backes[1], Rudi Balling[3], Fabian Kern[1], Rejko Krüger[3], Frank Lammert[4], Nicole Ludwig[5], Benjamin Meder[6], Bastian Fromm[7] ,Walter Maetzler[8], Daniela Berg[8], Kathrin Brockmann[9], Christian Deuschle[9], Anna-Katharina von Thaler[9], Gerhard W. Eschweiler[10], Sofiya Milman[11], Nir Barziliai[11], Matthias Reichert[4], Tony Wyss-Coray[2], Eckart Meese[5], Andreas Keller[1,2,12,*]

Supplementary Information

Supplementary Figure 1

Supplementary Figure 2

Supplementary Figure 3

Supplementary Figure 4

Supplementary Figure 5

Supplementary Figure 6

Supplementary Fig. 1

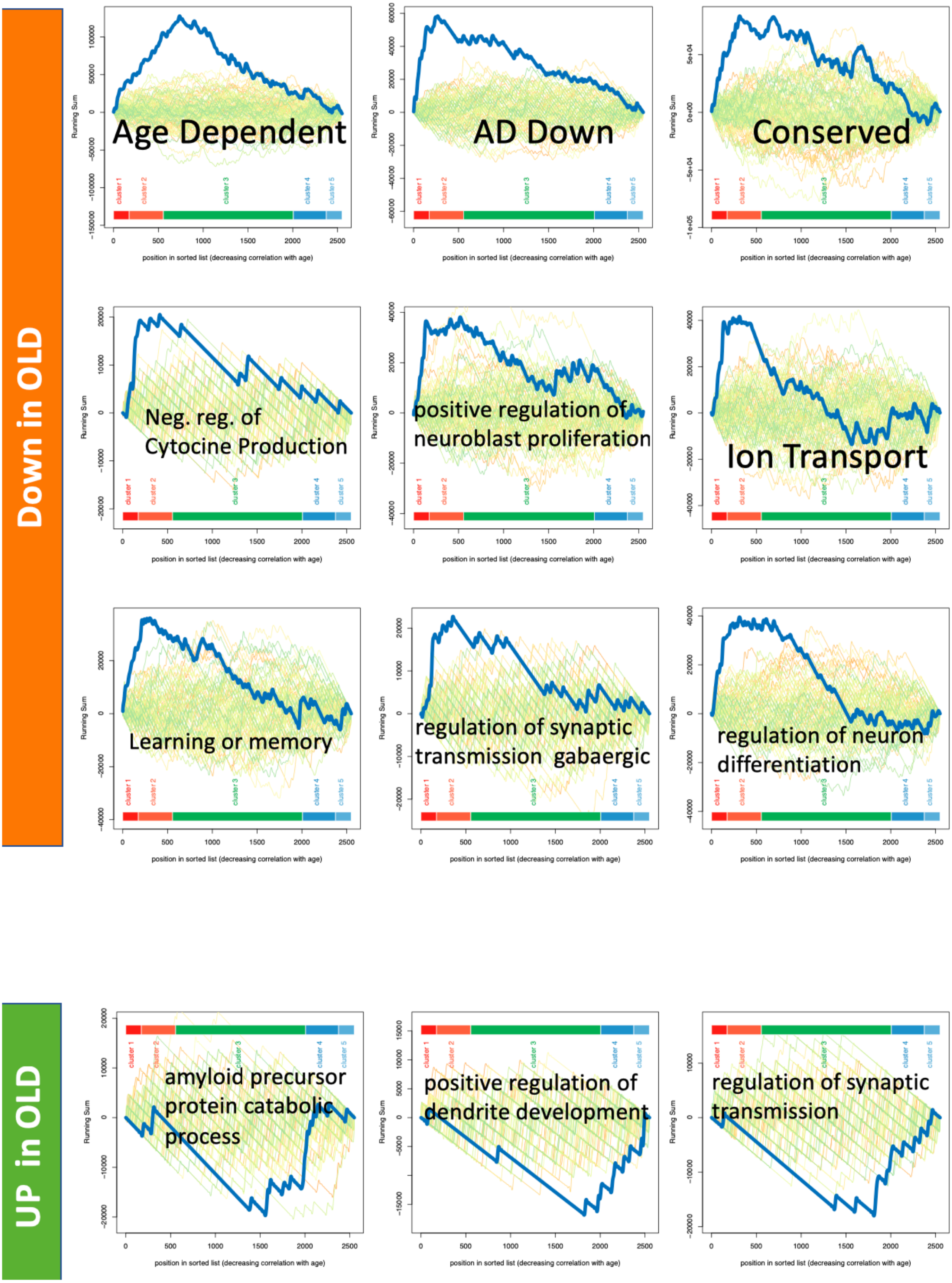

Supplementary Fig. 2

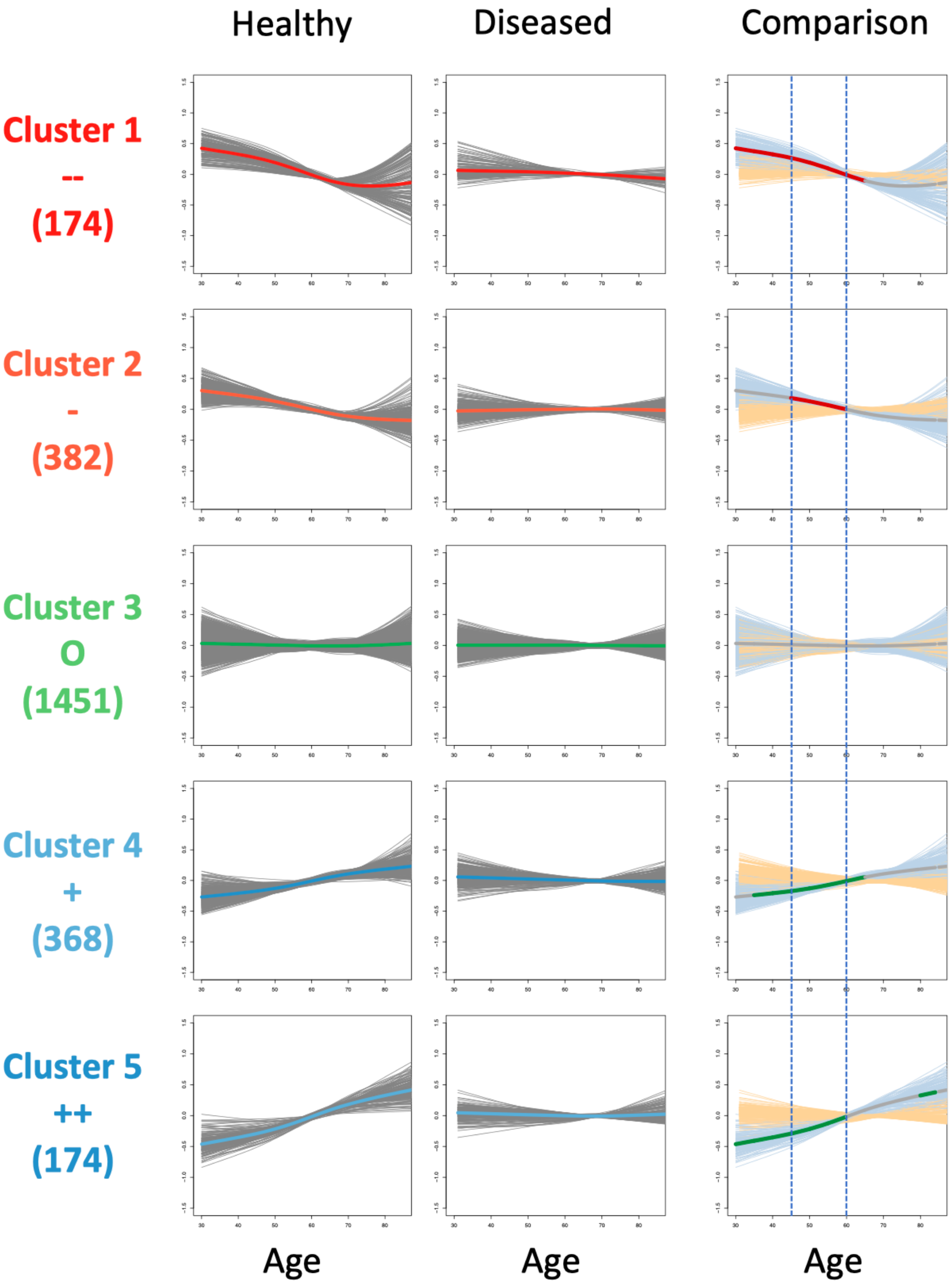

Supplementary Fig. 3A

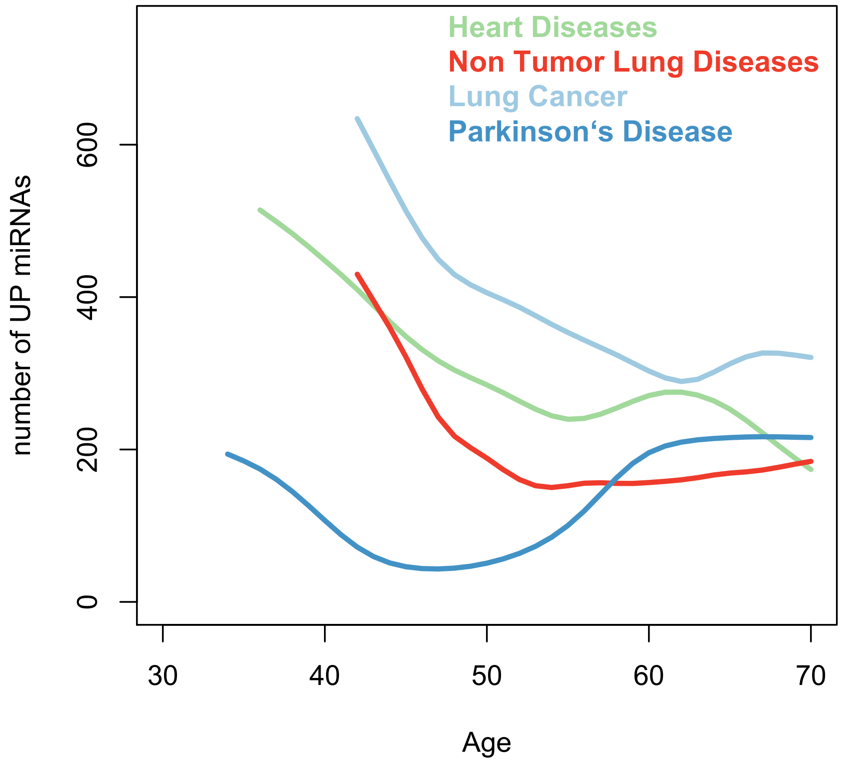

Supplementary Fig. 3B

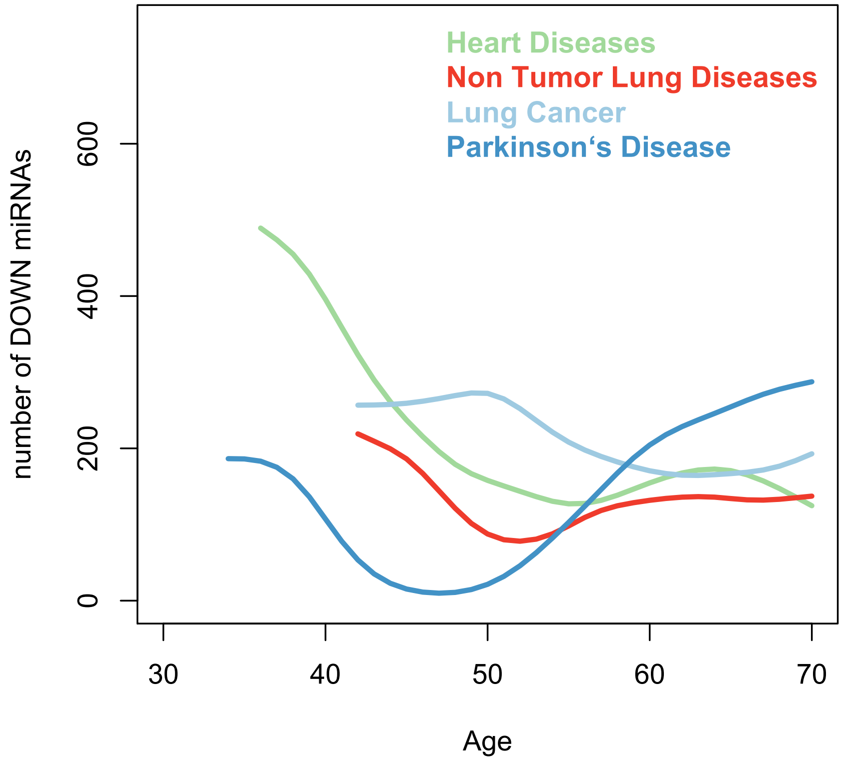

Supplementary Fig. 4

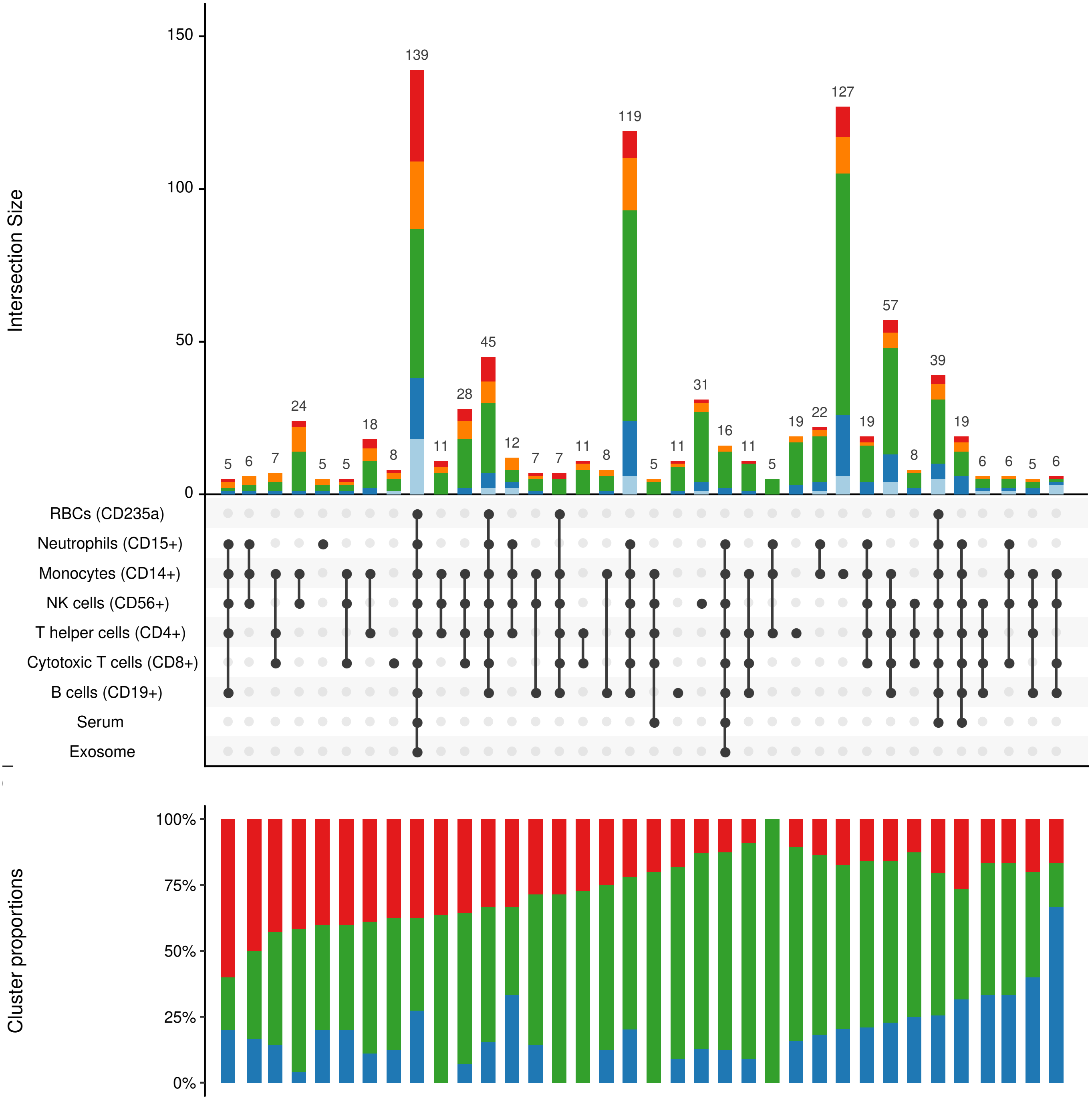

Supplementary Fig. 5

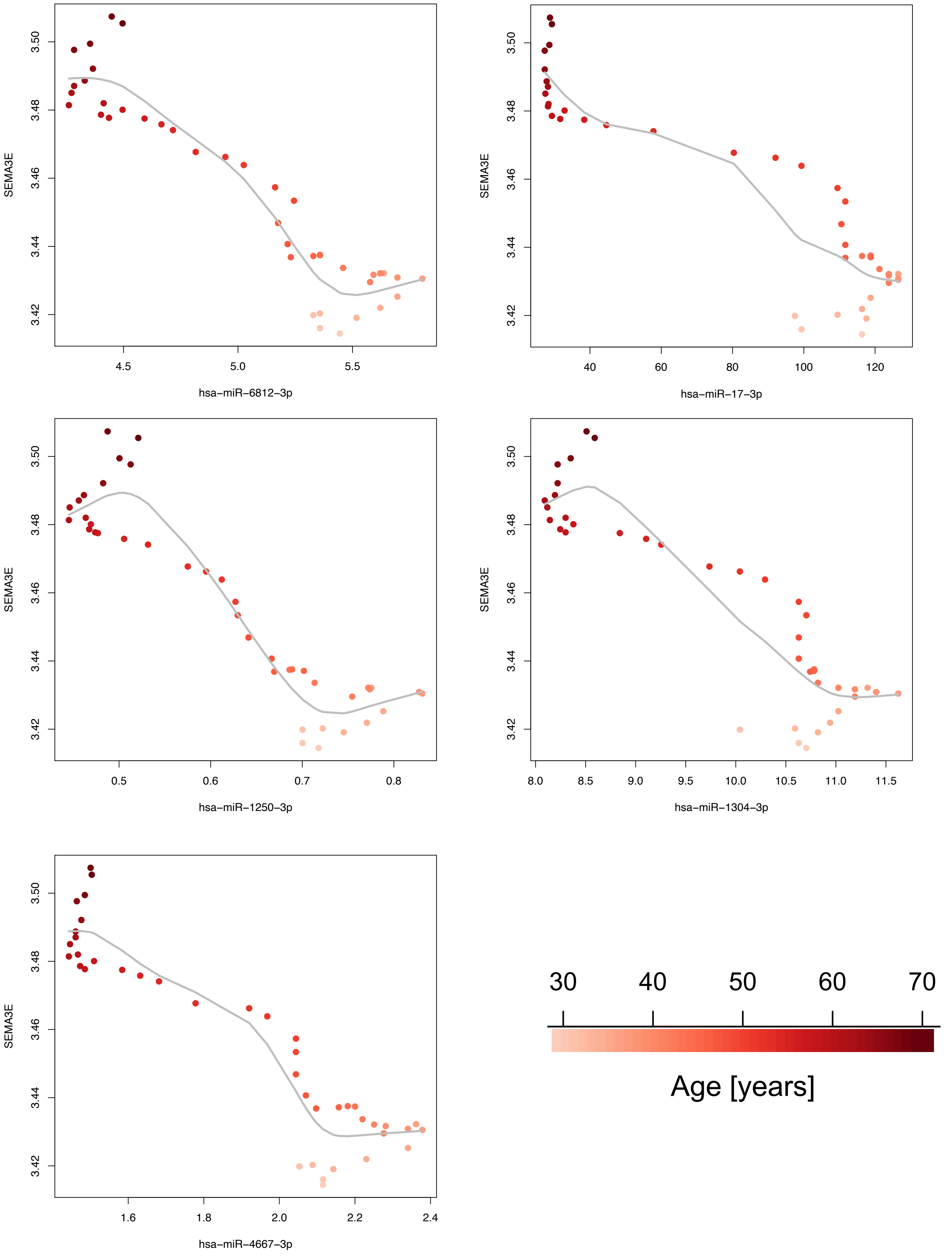

Supplementary Fig. 6

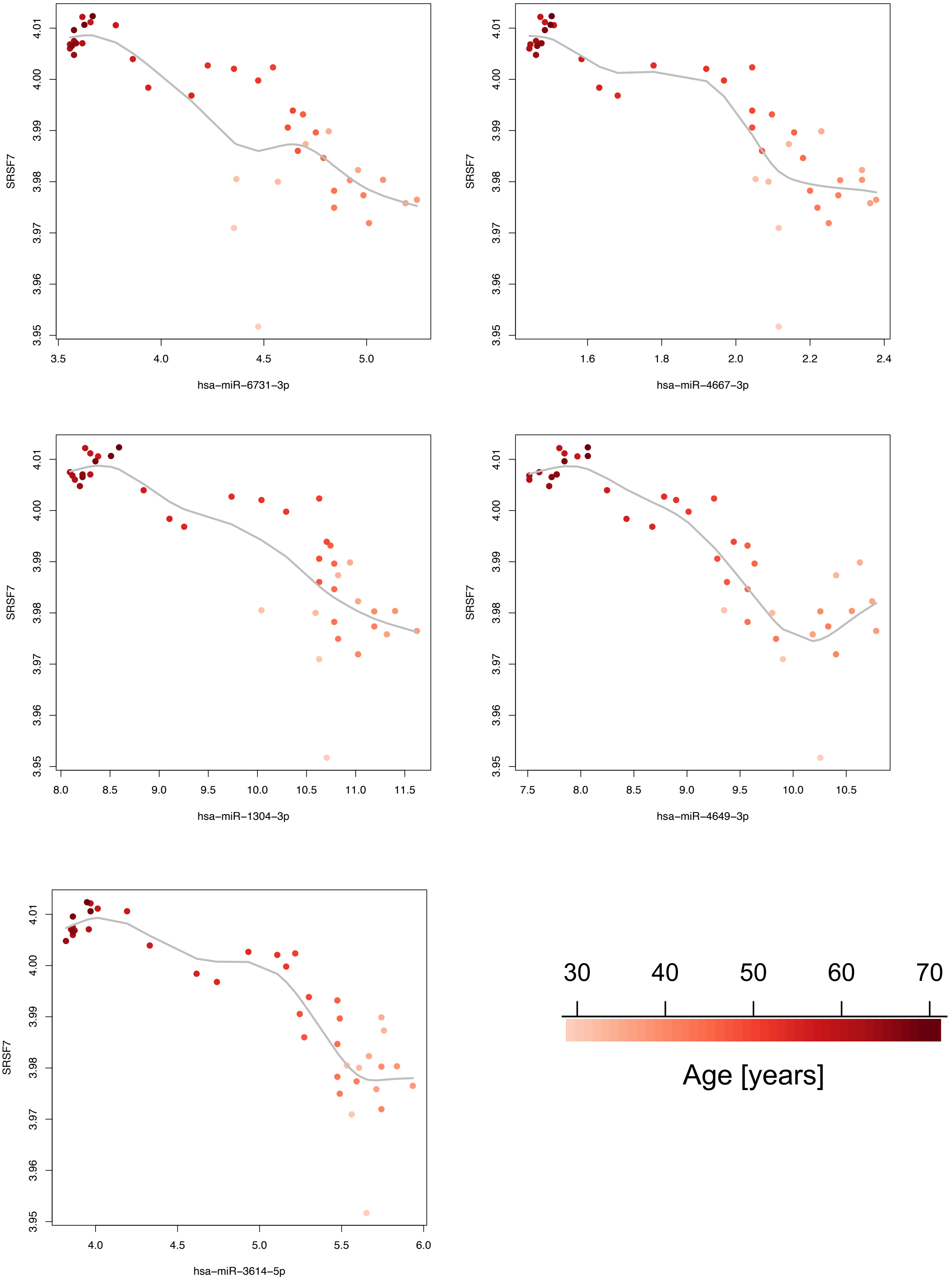

Supplement: Supplementary file 1 — Supplementary Information [file 41467_2020_19665_MOESM1_ESM.pdf]
