# Supplementary material for: Common diseases alter the physiological age-related blood microRNA profile
Source: Nat Commun. 2020 Nov 24;11:5958. doi: 10.1038/s41467-020-19665-1 (PMC7686493; doi:10.1038/s41467-020-19665-1)
Supplement: Supplementary file 3 — Description of Additional Supplementary Files [file 41467_2020_19665_MOESM3_ESM.pdf]

Description of Additional Supplementary Files

File Name: Supplementary Data 1
Description: Sample number, RIN value indicative of the RNA quality and disease annotation for all samples.

File Name: Supplementary Data 2
Description: Cluster association for each miRNA

File Name: Supplementary Data 3
Description: Pathway associations for miRNAs. The pathway database, the actual pathway and the direction of enrichment are presented. Next the p-value computed by a running sum statistics (GSEA) that has been adjusted for multiple testing by the Benjamini-Hochberg approach is given. The 5th column denotes the number of miRNAs in this category and the 6th column enumerates those miRNAs.

File Name: Supplementary Data 4
Description: For each miRNA, the linear and nonlinear correlation is provided. The 4th column represents the difference between linear and non-linear correlation and the 5th column contains the distance from the spline. In the last column, the direction (up/down) of the miRNA is provided.

File Name: Supplementary Data 5
Description: Correlation of the percentage of 5' expression with age (2nd column) absolute 3' expression (3rd column), absolute 5' expression (4th column) and the p-value of the percentage of 5' correlation with age (5th column). The p-values have been computed via the asymptotic t approximation.

File Name: Supplementary Data 6
Description: For each miRNA we compute the p-value for the correlation with age in controls (column 2) and patients (column 3) via the asymptotic t approximation. Column 4 contains a Boolean flag that is one only if the miRNA is significant following Benjamini-Hochberg adjustment only in controls but not in patients.

File Name: Supplementary Data 7
Description: Mapping of each miRNA to the SOM Grid Cell.

File Name: Supplementary Data 8
Description: For each miRNA (rows) the effect size in each disease as compared to controls and each age window are presented in detail. Values are NA if the window did not contained sufficient number of samples to compute the effect size.
