# Supplementary material for: Common diseases alter the physiological age-related blood microRNA profile
Source: Nat Commun. 2020 Nov 24;11:5958. doi: 10.1038/s41467-020-19665-1 (PMC7686493; doi:10.1038/s41467-020-19665-1)
Supplement: Supplementary file 12 — Reporting Summary [file 41467_2020_19665_MOESM12_ESM.pdf]

natureresearch

Corresponding author(s): Andreas Keller

Last updated by author(s): Oct 2, 2020

Reporting Summary

Nature Research wishes to improve the reproducibility of the work that we publish. This form provides structure for consistency and transparency in reporting. For further information on Nature Research policies, seeAuthors & Referees and theEditorial Policy Checklist .

Statistics

For all statistical analyses, confirm that the following items are present in the figure legend, table legend, main text, or Methods section.

n/a	Confirmed	
☐	☒	The exact sample size (*n*) for each experimental group/condition, given as a discrete number and unit of measurement
☐	☒	A statement on whether measurements were taken from distinct samples or whether the same sample was measured repeatedly
☐	☒	The statistical test(s) used AND whether they are one- or two-sided<br>*Only common tests should be described solely by name; describe more complex techniques in the Methods section.*
☐	☒	A description of all covariates tested
☐	☒	A description of any assumptions or corrections, such as tests of normality and adjustment for multiple comparisons
☐	☒	A full description of the statistical parameters including central tendency (e.g. means) or other basic estimates (e.g. regression coefficient) AND variation (e.g. standard deviation) or associated estimates of uncertainty (e.g. confidence intervals)
☐	☒	For null hypothesis testing, the test statistic (e.g. *F*, *t*, *r*) with confidence intervals, effect sizes, degrees of freedom and *P* value noted<br>*Give P values as exact values whenever suitable.*
☒	☐	For Bayesian analysis, information on the choice of priors and Markov chain Monte Carlo settings
☒	☐	For hierarchical and complex designs, identification of the appropriate level for tests and full reporting of outcomes
☐	☒	Estimates of effect sizes (e.g. Cohen's *d*, Pearson's *r*), indicating how they were calculated

Our web collection on statistics for biologists contains articles on many of the points above.

Software and code

Policy information about availability of computer code

Data collection	All data have been generated within the study, there was no software used to e.g. extract data from public databases or similar.
Data analysis	The data anylsis has been performed using the R software for statistical computation (R 3.3.2 GUI 1.68 Mavericks build (7288)). The following packages were used: ROC, RColorBrewer, preprocessCore, tsne, effsize, UpSetR, kohonen, fmsb, igraph

For manuscripts utilizing custom algorithms or software that are central to the research but not yet described in published literature, software must be made available to editors/reviewers. We strongly encourage code deposition in a community repository (e.g. GitHub). See the Nature Research guidelines for submitting code & software for further information.

Data

Policy information about availability of data

All manuscripts must include a data availability statement. This statement should provide the following information, where applicable:

- Accession codes, unique identifiers, or web links for publicly available datasets
- A list of figures that have associated raw data
- A description of any restrictions on data availability

The raw microarray measurements are freely available for any scientific purpose upon request as Ecxel Table and Tab Delimited Text file (110 MB) to data@ccb.uni-saarland.de. The use of the data for commercial purposes is prohibited.

Field-specific reporting

Please select the one below that is the best fit for your research. If you are not sure, read the appropriate sections before making your selection.

October 2018

Life sciences study design

All studies must disclose on these points even when the disclosure is negative.

Sample size	Based on previous studies on microRNAs we assume cohort sizes of 20-60 samples to be sufficient for reaching adequate power. A post-hoc power calculation on the actual result in this study demonstrate that for all diseases a power of 1 is reached at an alpha level of 0.05 for the most significant markers.
Data exclusions	In this study, we processed data from ntotal=4,433 whole blood samples. We excluded 40 individuals (0.9%) because of insufficient data quality or missing clinical or demographic information. The final cohort consists thus of 4,393 samples.
Replication	To test the reproducibility across time, we continuously measured 1 individual 41 times uniformly distributed more than 1000 consecutive measurements. An average Pearson correlation of 0.995 (95% CI, 0.9948-0.9952) was calculated (Fehmann et al, 2020), validating the technical stability of the system.
Randomization	Randomization did not apply to this study as participants were not assigned to treatment groups.
Blinding	The age and gender of the patients has been only made available after having had access to the already measured data. For the majority of samples the clinical information was blinded. Exceptions are the cohort studies, for example, samples from CosyCoNet are known to be from COPD patients making blinding impossible.

Reporting for specific materials, systems and methods

We require information from authors about some types of materials, experimental systems and methods used in many studies. Here, indicate whether each material, system or method listed is relevant to your study. If you are not sure if a list item applies to your research, read the appropriate section before selecting a response.

Materials & experimental systems

n/a	Involved in the study	
☒	☐	Antibodies
☒	☐	Eukaryotic cell lines
☒	☐	Palaeontology
☒	☐	Animals and other organisms
☐	☒	Human research participants
☒	☐	Clinical data

Methods

n/a	Involved in the study	
☒	☐	ChIP-seq
☒	☐	Flow cytometry
☒	☐	MRI-based neuroimaging

Human research participants

Policy information about studies involving human research participants

Population characteristics	The population consists of 4,433 whole blood samples. We excluded 40 individuals (0.9%) because of insufficient data quality or missing clinical or demographic information. The final cohort consists thus of 4,393 samples. These include unaffected controls (nHC=1,334), Parkinson's Disease (nPD=944), heart diseases (nHD=607), non-tumor lung diseases (nNTLD=586), lung cancer (nLC=517), and other diseases (nOD=405). The age and gender have not been used as covariates since they are the main esponse variable and the study aim was to investigate age and gender effects on biomarker profiles.
Recruitment	The indviduals have been included from basically two study types, cohort studies (TREND from Tuebingen and the multi-centric COSYCONET study) and case-control studies (all other samples). We did not observe any global difference between samples from the two stuy types since identical collection protocols were used. The study is a retrospective study.
Ethics oversight	The IRB of Saarland university (Ärztekammer des Saarlandes) approved the study.

Note that full information on the approval of the study protocol must also be provided in the manuscript.
